# Serological Assays for Assessing Postvaccination SARS-CoV-2 Antibody Response

**DOI:** 10.1128/Spectrum.00733-21

**Published:** 2021-09-29

**Authors:** Sally A. Mahmoud, Subhashini Ganesan, Shivaraj Naik, Safaa Bissar, Isra Al Zamel, KN Warren, Walid A. Zaher, Gulfaraz Khan

**Affiliations:** a Biogenix Lab G42, Masdar City, Abu Dhabi, United Arab Emirates; b G42 Healthcare, Masdar City, Abu Dhabi, United Arab Emirates; c College of Medicine and Health Sciences, United Arab Emirates University, Al Ain, Abu Dhabi, United Arab Emirates; Montefiore Medical Center and Albert Einstein College of Medicine

**Keywords:** SARS-CoV-2, serological assays, COVID-19, ELISA, CLIA

## Abstract

Serological assays for measuring severe acute respiratory syndrome coronavirus 2 (SARS-CoV-2) antibodies have crucial applications in the control and surveillance of the current COVID-19 pandemic. A large number of such assays have been developed and are now commercially available. However, there are limited studies evaluating the performance of these tests. We evaluated the performances of the following six commercially available serological assays for detecting SARS-CoV-2 antibodies: (i) Genscript cPass surrogate virus neutralization test (Genscript cPass), (ii) Diasorin-SARS-CoV-2 S1/S2 IgG detection (Diasorin-S1/S2 IgG), (iii) Alinity SARS-CoV-2 IgG II (Alinity IgG II), (iv) Diasorin-SARS-CoV-2 TrimericS IgG (Diasorin-TrimericS IgG), (v) Roche Elecsys anti-SARS-CoV-2-cobas (Roche Elecsys), and (vi) AESKU enzyme linked immunosorbent assay (AESKULISA). The results of these tests were compared against the gold standard plaque reduction neutralization test (PRNT). Roche Elecsys had the highest sensitivity, and the Genscript cPass had the highest specificity. Diasorin-TrimericS IgG had the best overall performance with the highest agreement with the PRNT results. Parallel testing of Genscript cPass with Diasorin-TrimericS IgG and Diasorin-S1/S2 IgG had the optimum performance. Based on the receiver operating characteristic (ROC) curve, lowering the cutoff from 30% to 20% in the Genscript cPass significantly increased the sensitivity and the overall agreement with the PRNT results. Commercially available serological assays are good alternatives to the standard PRNT. However, further studies on larger sample numbers are required for optimization of the assay cutoff values and for evaluation of cost effectiveness.

**IMPORTANCE** Commercial serological assays for severe acute respiratory syndrome coronavirus 2 (SARS-CoV-2) are now widely available. This study adds new knowledge regarding the optimization of these assays for evaluating postvaccination antibodies status. It highlights the positive and negative aspects of each assay in terms of sensitivity, specificity, and positive and negative predictive values, compared to the gold standard neutralization test. When using serological assays to assess postvaccine immune status, a balance of all parameters needs to be considered and not simply the high specificity. This balance is particularly relevant in the current situation where countries are aiming to mass vaccinate their populations and bring this pandemic under control. Assays with good sensitivity will have a lower percentage of false negatives and thus provide confidence for vaccination. Understanding the strengths and limitations of commercially available serological assays is important, not only for better application of these tests but also to understand the immune response and the duration of protection postvaccination.

## INTRODUCTION

Severe acute respiratory syndrome coronavirus 2 (SARS-CoV-2), the cause of the current COVID-19 pandemic, was first identified in a small group of patients in Wuhan, China, in December 2019 (1). The virus, believed to be of zoonotic origin (2), quickly adapted to the human host and spread rapidly across the globe (3). By 30 January 2020, WHO declared it a public health emergency of international concern. In spite of countries implementing various containment and mitigation measures, including travel restrictions, extensive lockdowns, social distancing, and mask wearing (4–6), the virus continued to spread and caused an unprecedented level of morbidity and mortality (7). Global efforts focused on establishing rapid and reliable diagnostic tests, finding effective treatments, and developing vaccines for prevention (8–10).

More than 18 months into the pandemic, we have several approved vaccines being used widely for mass vaccination (11–13). During the study period, the UAE population was vaccinated by an inactivated vaccine against SARS-CoV-2 created by the Beijing Institute of Biological Products called BBIBP-CorV. This vaccine works by stimulating the immune system to make antibodies against the SARS-CoV-2 coronavirus. The vaccine was prepared by multiplication of SARS-CoV-2 WIV04/HB02 strain in African green monkey kidney cells grown in bioreactor tank and produced large stocks of the coronaviruses. The virus was then inactivated by a chemical called beta-propiolactone and the inactivated viruses was mixed with a tiny amount of an aluminum-based compound called an adjuvant. Adjuvants stimulate the immune system to boost its response to a vaccine. The vaccine stimulates the immune system to produce antibodies that target the spike protein of the SARS-CoV-2 virus that can prevent the virus from entering cells and thereby preventing SARS-CoV-2 infections (14). Identification and quantification of the antibody production against SARS-CoV-2 within individuals is important for assessing the effectiveness and longevity of vaccines, as well as for informing national and international policy on vaccination strategies. There are several serological testing platforms that are used to evaluate the antibody status the FDA has issued emergency authorization for ELISA, lateral flow immunoassay, and microsphere immunoassay (15). These tests measure antibodies to the SARS-CoV-2 nucleocapsid N protein or the spike protein S. Since the virus enters the cell via binding of the S protein to its cell surface receptor ACE_2_ measuring antibodies to the S protein is of particular importance in preventing disease manifestation (16, 17). A meta-analysis on serological assays for detecting antibodies against SARS-CoV-2 indicated that assays using the S antigen and testing for IgG antibodies have better sensitivity than the N antigen- and IgM-based tests (18). This is a particularly salient marker of efficacy for vaccines like the inactivated BBIBP-CorV vaccine because they stimulate the immune system to produce antibodies against the spike (S) protein on the surface of the virus; therefore, this study looks at the anti-S antibodies against the SARS-CoV-2 virus. However, not all spike-binding antibodies are functional or block viral infection. Hence, they do not necessarily indicate the functional measure of the antibody. Ideally, tests should measure the neutralizing antibodies, which implicate protection from infection (19, 20). The gold standard for measuring neutralizing antibodies is the plaque reduction neutralization test (PRNT). PRNT is however not practical for large-scale testing, as it requires skilled manpower, high-level biohazard security (biosafety level 3 [BSL-3]), and a long turnaround time of 5 days (21, 22).

Therefore, to address this gap, a number of commercial serological assays have been developed and are now available in the market. A meta-analysis performed on these serological assays for detecting antibodies against SARS-CoV-2 have indicated that assays using the S antigen and testing for IgG antibodies have better sensitivity than the N antigen- and IgM-based tests (23). It is important to understand the strengths and limitations of commercially available serological assays, not only for the appropriate application of these tests but also to better understand the immune response and the duration of protection after vaccination. This is particularly relevant now, as mass vaccination is being rolled out. These serological assays have enormous potential application in handling the current pandemic, both at individual and population level. At the individual level, it can help differentiate recent and past COVID-19 infections, check immune status postvaccination to determine the need for booster doses, and identify and optimize vaccine intervals. At the population level, serological testing can help unravel the epidemiology of the SARS-CoV-2 pandemic, assess the protective status of the population, and, thereby, help public health experts make recommendations regarding travel, social distancing, and lifting restrictions (24). Hence this study aims to evaluate six commercially available serological assays using serum samples from pre and postvaccination for SARS-CoV-2 and by comparing the results of these assays with the reports based on neutralizing titers as measured by PRNT using the same serum samples.

## RESULTS

The study included a total of 125 serum samples. The demographics of the participants showed that they were 70% male and 30% female, aged between 16 and 77 years; the mean age of the participants was 42.41 ± 13.71. The gold standard PRNT was performed on all of the samples. Based on the PRNT, 69 samples were positive and 56 samples were negative for SARS-CoV-2. The same samples were also subjected to evaluation using the other six serological assays. However, due to a lack of sufficient sample volume, some of the serological assays were not performed on all of the 125 samples. Table S2 shows the number of samples tested using each assay and the results for each. The borderline/equivocal results that were above the cut-off values for positive reports were considered positive for SARS-CoV-2.

Roche Elecsys had the highest sensitivity (100%), followed by Alinity IgG II (98.1%) and AESKULISA (92.3%). Genscript cPass had the highest specificity (94.6%), followed by Diasorin-S1/S2 IgG detection (94.1%) and Diasorin-Trimeric S IgG (90%) (Table 1).

**TABLE 1:** Performance indicators for the 6 serological assays

Assay (*n*)	% (95% CI) by indicator				
	Sensitivity	Specificity	PPV	NPV	Overall agreement with PRNT results
Genscript-cPass (125)	71.07 (58.8–81.3)	94.64 (85.1–98.8)	94.23 (84.1–98.8)	72.60 (60.9–82.4)	81.6 (73.7–87.9)
Diasorin-S1/S2 IgG (104)	75.47 (61.7–86.2)	94.12 (83.8–98.8)	93.02 (80.9–98.5)	78.69 (66.3–88.1)	84.61 (76.2–90.9)
Alinity IgG II (104)	98.11 (89.9–99.9)	70.59 (56.1–82.5)	77.61 (65.8–86.9)	97.30 (85.8–99.9)	84.62 (76.22–90.94)
Diasorin-TrimericS IgG (103)	84.91 (72.4–93.2)	90 (78.1–96.6)	90 (78.1–96.6)	84.91 (72.4–93.2)	87.37 (79.4–93.1)
Roche Elecsys (96)	100 (92.6–100)	41.67 (27.6–56.7)	63.16 (51.3–73.9)	100 (83.1–100)	70.83 (60.7–79.7)
AESKULISA (99)	92.31 (81.5–97.9)	72.34 (57.4–84.4)	78.69 (66.3–88.1)	89.47 (75.2–97.1)	82.82 (73.9–89.7)

Genscript cPass surrogate virus neutralization test being a functional test was combined with a quantitative test in series testing, it showed that Genscript cPass in parallel testing with Diasorin-S1/S2 IgG or Diasorin-SARS-CoV-2 TrimericS IgG T2 (Diasorin-TrimericS IgG) had the optimum sensitivity (92.8%–95.6%) and specificity (85.1%–89.1%) and the results can be obtained in 3 hours (Table 2). Genscript cPass surrogate virus neutralization test kit in serial testing with Diasorin-SARS-CoV-2 S1/S2 IgG showed the maximum specificity (99.69%) (Table 2).

**TABLE 2:** Genscript cPass surrogate virus neutralization test in parallel and series testing with other serological assays

Assay with Genscript cPass	Parallel testing (%)		Series testing (%)	
	Sensitivity	Specificity	Sensitivity	Specificity
Diasorin-S1/S2 IgG	92.86	89.07	53.53	99.69
Alinity IgG II	99.46	66.8	69.65	98.41
Diasorin-TrimericS IgG	95.63	85.17	60.27	99.46
Roche Elecsys	100	39.72	71	96.9
AESKULISA	97.77	68.46	65.54	98.52

**FIG 1:**
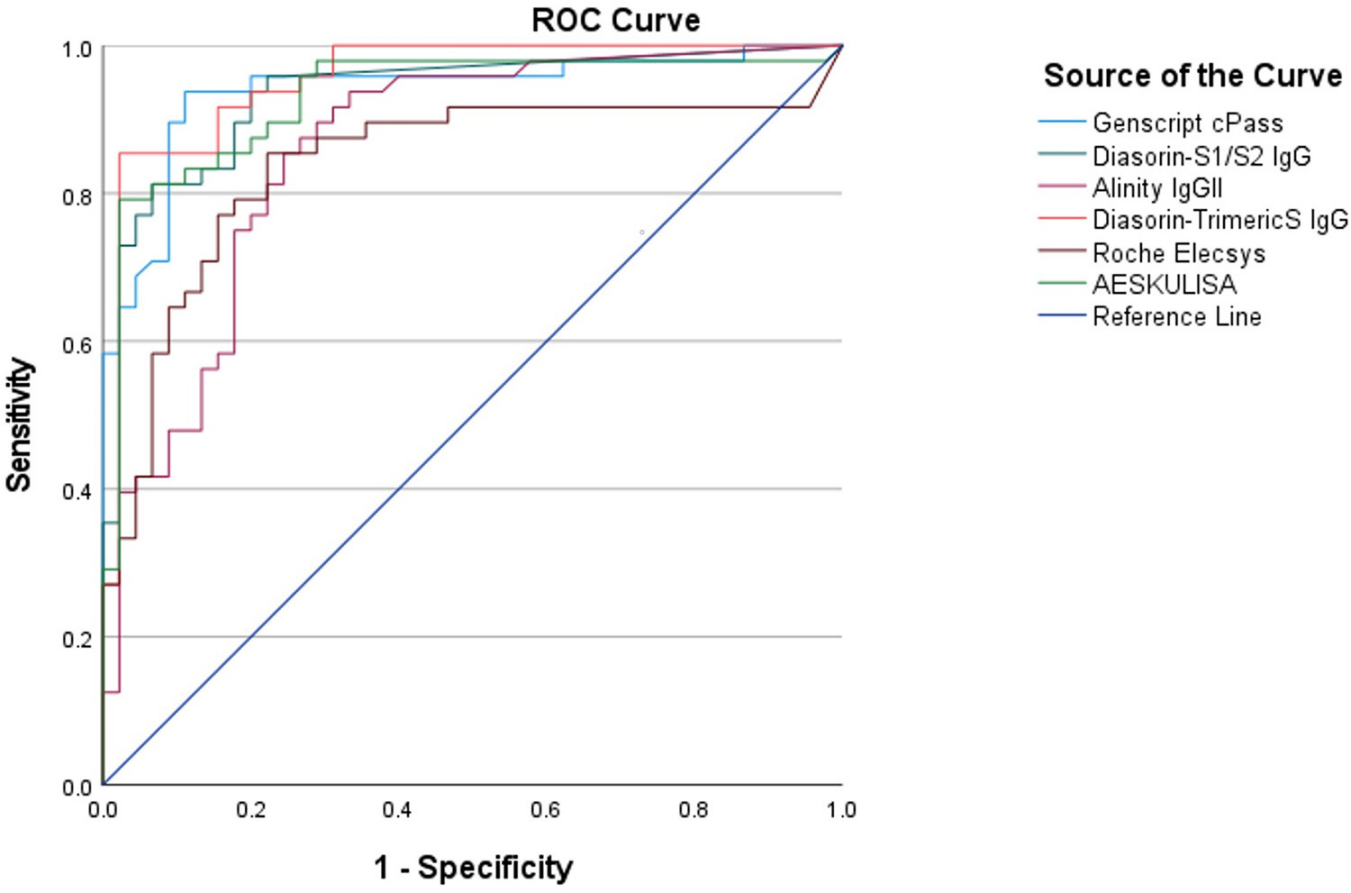
ROC curves for the serological assays.

ROC curves demonstrating the best ability to differentiate positive and negative results in comparison to the PRNT results were plotted for all the serological assays and the area under the curve (AUC) was largest for Diasorin-TrimericS IgG test (0.953), followed by Genscript cPass (0.939) and Diasorin S1/S2 IgG (0.935) assays (Fig. 1; Table S3 and S4). Cut-off values for each test were estimated based on the ROC optimum cut-off. Genscript cPass, Diasorin-TrimericS IgG, and Roche Elecsys showed different cut-offs than what was recommended by the manufacturers. From the new adopted ROC cut-offs, we calculated the sensitivity, specificity, and overall agreement with the results of the PRNT assay (Table 3).

**TABLE 3:** Optimum cutoffs based on the ROC curves and their performances^*a*^

Assay (*n*)	Cutoff	Sensitivity	Specificity	Overall agreement (%)
Genscript cPass (125)	Manufacturer’s cutoff, 30%	71%	94.23%	81.6%
	Based on ROC cutoff, 20%	92.75%	87.5%	90.4%
	*P* value	<0.001	0.065	<0.05
Diasorin-TrimericS IgG (103)	Manufacturer’s cutoff, 33.8 BAU/ml	84.91%	90%	87.37%
	Based on ROC cutoff, 40 BAU/ml	84.90%	96%	90.29%
	*P* value	0.998	0.092	0.506
Roche Elecsys (96)	Manufacturer’s cutoff, 0.8 U/ml	100%	41.67%	70.83%
	Based on ROC cutoff, 5 U/ml	93.75%	70.83%	82.29%
	*P* value	<0.05	<0.001	0.0616

a The other three tests showed ROC-based cutoffs similar to the manufacturer’s values.

**FIG 2:**
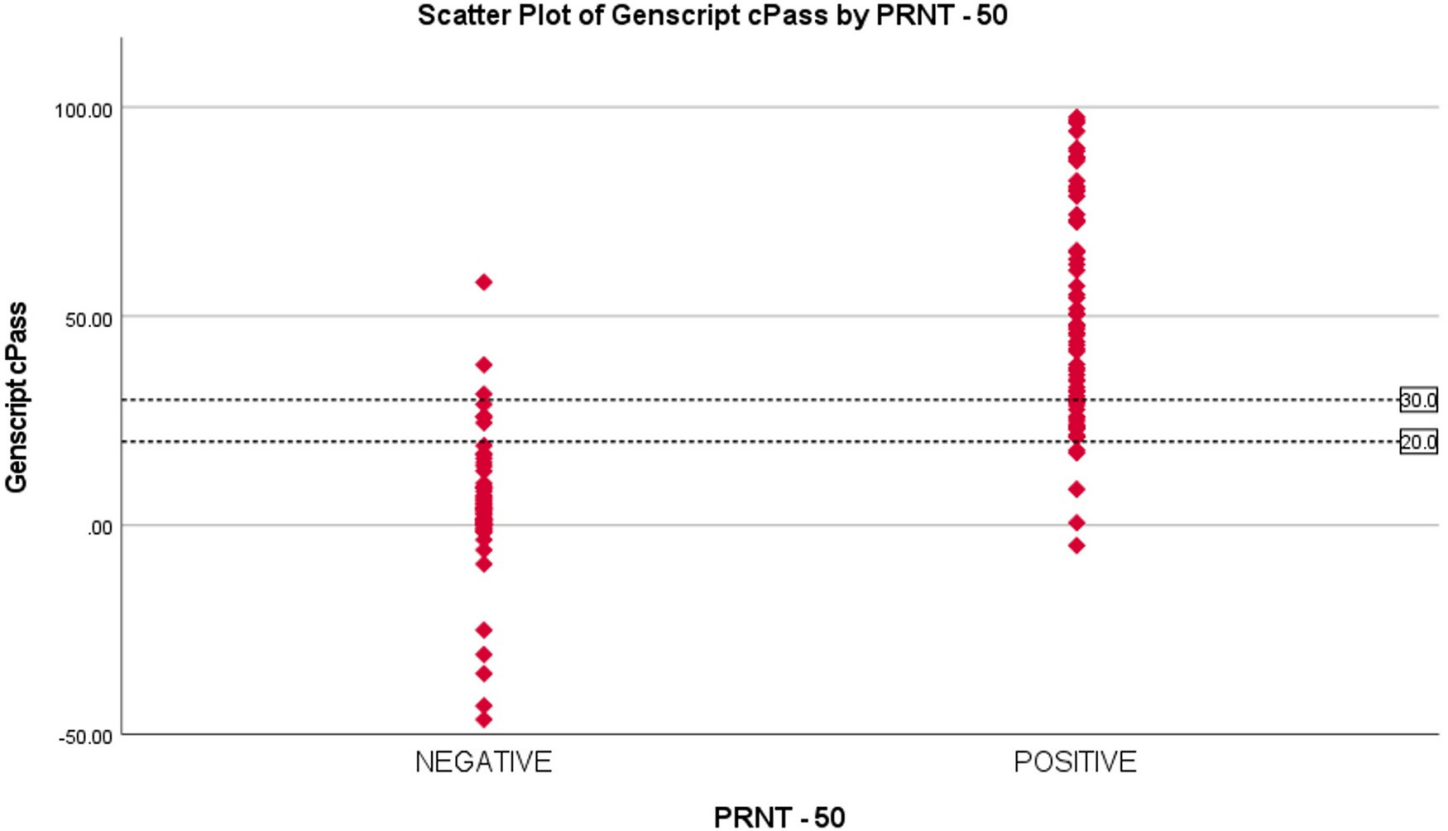
Scatterplot of percentage inhibition using Genscript cPass test based on results of the PRNT.

The ROC curve showed 20% as an optimum cut-off for Genscript cPass. 20 samples that were reported positive by PRNT were reported negative by Genscript cPass. 13 out of these 20 samples (65%) had inhibition percentage of ≥ 20%. This demonstrates the fact that 65% of the positive samples are missed by Genscript cPass by increasing the cut-off to 30% (Fig. 2).

Similarly, in Diasorin-TrimericS IgG by raising the cut-off to 40 BAU/ml, 3 out of 13 (23%) samples that showed discrepnacies with the PRNT reports were correctly reported as negative, therby increasing the specificity and overall agreement with PRNT.

In Roche Elecsys by raising the cut-off from 0.8 to 5U/ml, our study found that 14 out of 28 (50%) samples that was initially reported as positive by the test were correctly reported as negative.

Our results also showed that 2 serum samples that were identified as positive by all the serological assays were reported negative by the PRNT assay at <1:20 dilution; however, we did not find any sample with a positive PRNT result reported negative by all the other serology assays.

Cohen’s kappa coefficient was calculated to see the agreement between the assays and PRNT and we found that Diasorin-TrimericS IgG had the maximum kappa coefficient value (0.750), showing highest agreement with PRNT (Table S5).

Receiver operating characteristic (ROC) curves demonstrating the best ability to differentiate positive and negative results in comparison to the PRNT results were plotted for all the serological assays and the AUC was largest for Diasorin-TrimericS IgG test (0.953), followed by Genscript cPass (0.939) and Diasorin S1/S2 IgG (0.935) assays (Fig. 1) (Table S3 and Table S4). Cut-off values for each test were estimated based on the ROC optimum cut-off. Genscript cPass, Diasorin-TrimericS IgG, and Roche Elecsys showed different cut-offs than what was recommended by the manufacturers. From the new adopted ROC cut-offs, we calculated the sensitivity, specificity, and overall agreement with the results of the PRNT assay (Table 3).

The ROC curve showed 20% as an optimum cut-off for Genscript cPass. 20 samples that were reported positive by PRNT were reported negative by Genscript cPass. 13 out of these 20 samples (65%) had inhibition percentage of ≥ 20%. This demonstrates the fact that 65% of the positive samples are missed by Genscript cPass by increasing the cut-off to 30%. (Fig. 2).

Similarly, in Diasorin-TrimericS IgG by raising the cut-off to 40 BAU/ml, 3 out of 13 (23%) samples that showed discrepnacies with the PRNT reports were correctly reported as negative, therby increasing the specificity and overall agreement with PRNT.

In Roche Elecsys by raising the cut-off from 0.8 to 5U/ml, our study found that 14 out of 28 (50%) samples that was initially reported as positive by the test were correctly reported as negative.

## DISCUSSION

This study compared six immunoassays for the detection of IgG antibodies against SARS-CoV-2 with the standard viral neutralization test PRNT. Our findings indicated that the Genscript cPass test had the lowest sensitivity but a high specificity and positive predictive value (PPV). Other studies which have evaluated Genscript cPass reported the test to have high sensitivity (25, 26). This disparity may be due to the fact that these studies used the cutoff as 20% inhibition, which was later changed according to FDA recommendation to 30% (27). A study that compared Genscript cPass to PRNT, using 30% as the cutoff, reported the sensitivity to be in the range of 77% to 100% and specificity to be 95% to 100% (28). These findings are similar to our study when 30% cutoff was used.

The ROC curve-adapted cutoff for the Genscript cPass test was around 20% inhibition, and when this value was used as a cutoff, the sensitivity increased to 92.31% and the overall agreement with the PRNT assay increased to 90.4%. Using Chi square, the difference was statistically significant, suggesting 20% inhibition as a more optimum cutoff for the test. Another study that evaluated the Genscript cPass test also suggested that the test might require specific cutoffs with respect to patient ethnicity, geographical background, and prevalence of COVID-19 infection. It also stated that the introduction of an equivocal range with repeat testing within the range of 18% to 22% can reduce the false-positive results (29).

However, according to the ROC values, raising the cutoffs for Diasorin-TrimericS IgG and Roche Elecsys tests increased the agreement with the PRNT results, but the difference was not significant.

Studies suggest the necessity for a revision of cutoff values provided by manufacturers, as most of the assay validations are done on a small sample size and among specific ethnic or regional groups (30). Therefore, more evaluation studies and optimum cutoffs need to be defined before these serological assays are used in large-scale testing for assessing the vaccination status of the population.

Roche Elecsys and Alinity IgG II demonstrated high sensitivity in this study. This agrees with the other studies, although, in contrast to our findings, these studies also reported high specificity (31–33). A meta-analysis on antibody tests for SARS-CoV-2 showed that tests using ELISA and CLIA-based methods performed better (18). Similar results were seen in our study for tests based on ELISA and CLIA, which showed high sensitivity. However, the sensitivities of the Genscript cPass virus neutralization test and Diasorin-S1/S2 IgG were low.

Alinity IgG II was evaluated in a study which compared antibodies in postvaccination patients to those in prepandemic serum samples, and they reported high sensitivity and a specificity of 100% (34). While our study showed that the sensitivity of Alinity IgG II was high, the specificity was low compared with specificity reported in reference (34). This finding might be because the latter study did not compare the assay with the PRNT method, as we have done, but instead with the RT-PCR assay.

Diasorin-TrimericS IgG had good sensitivity and specificity with the highest agreement with the PRNT results, which is in agreement with another study that evaluated the detection of circulating antibodies to SARS-CoV-2 using Diasorin (35). When a functional test like the Genscript cPass surrogate virus neutralization test was assessed in parallel with quantitative assays, the overall sensitivity was found to be higher. Optimum sensitivity and specificity are achieved when the Genscript cPass test is done in parallel with Diasorin-S1/S2 IgG detection. This combination of two antibody tests is being studied, and it shows that it increases the ability to better capture the positive results (36).

The CDC recommends serological tests with high sensitivity and specificity and tests detecting IgG or both IgG and IgM. This recommendation is because, currently, serological tests are recommended by CDC to only identify persons with previous infections or to identify resolving infections and to better understand the epidemiology of SARS-CoV-2 (37). However, these antibody tests can be very useful for assessing the immune response and the longevity of the antibodies developed postvaccination for COVID-19. This surveillance becomes essential particularly when examining vaccine efficiency and making recommendations on booster doses and the intervals for vaccination (19).

The WHO, in collaboration with Coalition for Epidemic Preparedness Innovations (CEPI) and the National Institute for Biological Standards and Control (NIBSC), has come up with the international standards for anti-SARS-CoV-2 immunoglobulins. These standards are crucial since vaccine developers have been using various immunoassays with different measuring units, which makes comparisons of immunogenicity difficult. Hence, with these recommendations, future studies can make the comparison of immunogenicity more standardized and reliable (38).

Most COVID-19 vaccinations target the virus spike protein S, which contains the receptor-binding domain (RBD) that binds to the host receptor ACE2 to gain entry into the cell (39). Thus, serological assays that detect antibodies against the S protein and the RBD serve as good candidates for evaluating vaccine response (19, 40) Additionally, a number of other parameters also need to be considered when performing serological assays for assessing postvaccine immune status, including timing after vaccination, number of doses, comorbidities, and age and sex of the patients (41, 42). This information is particularly relevant in the current situation in which global efforts are aimed at vaccinating as many people as possible to bring the pandemic under control (43). Indeed, some countries have already managed to vaccinate more than 50% of their eligible population. Thus, reliable, high-sensitivity serological assays will help to minimize false negatives and provide confidence to the public for vaccine efficacy. Thus, recommendations based on a balance of all these parameters are needed.

### Strengths.

Most evaluation studies compare serological assay results with RT-PCR reports. This study compares commercial serological assays using the same serum sample and by evaluating the results with the gold standard PRNT assay. Thus, this study reduces the biases and provides a standard comparison.

### Limitations.

The study did not consider demographic details of the study participants like age, sex, and comorbidities, which can influence antibody response to vaccination. The number of samples were limited and additional factors such, days post vaccination and the type of vaccine were not taken into consideration in this study, which could have provided further insights on the serological assay performances. This study was also limited by the sample ppavailability to evaluate every samples with all six assays alongside the gold standard PRNT.

### Conclusions.

Serological assays that are commercially available are efficient and show good agreement with the gold standard PRNT results. We recommend further studies on these serological assays with a large number of samples to help comprehensively evaluate the performance of these assays. Moreover, performing two tests in parallel can improve the sensitivity and provide a better alternative to the conventional PRNT assay. However, cost-effective evaluations will be required before drafting any recommendation. We also suggest optimizing cutoff values for these serological assays, based on prevalence, age, ethnic, and geographical variations. Recommendations based on the balance of all performance indicators, rather than just sensitivity or specificity, will help in the application of these serological assays in assessing postvaccination status.

## MATERIALS AND METHODS

The study evaluated six different methods for the detection of antibodies against SARS-CoV-2 in postvaccinated individuals and compared the results against the gold standard PRNT. The six different methods were as follows: (i) Genscript SARS-CoV-2 surrogate virus neutralization test (Genscript cPass), (ii) Diasorin-SARS-CoV-2 S1/S2 IgG detection (Diasorin-S1/S2 IgG), (iii) Alinity SARS-CoV-2 IgG II (Alinity IgGII), (iv) Diasorin-SARS-CoV-2 TrimericS IgG (Diasorin-TrimericS IgG), (v) Roche Elecsys anti-SARS-CoV-2-cobas (Roche Elecsys), and (vi) AESKU enzyme-linked immunosorbent assay (AESKULISA).

The PRNT is a gold standard serological test, which utilizes the ability of a specific antibody to neutralize a virus and, hence, prevent the virus from forming plaques in a cell monolayer. For this study, Vero E6 cells were grown to a confluent monolayer in a 6 well plate and for the positive control, serum sample collected from 60 patients 14-to-28 days postvaccination were pooled. For negative control, the viral stock dilution media, Dulbecco’s modification of Eagle medium (DMEM), was used. DMEM was preferred as a negative control over a negative serum sample to avoid interference from non-specific antibodies that may have been present in the serum. Results for each of the assays were read by comparing the plaque count in relation to the negative control and the interpretation of PRNT results were based on the dilution of serum identified at 50% reduction of the total plaque (virions) count in the negative control.

Optimizations of the viral culture and plaque assays were performed in the lab during which conditions, such as days of cell seeding before use in the PRNT, working viral dilution, volume of inoculum, time for the infection of cell monolayer with serum/virus mixture, percentage of agarose medium, and days post-incubation, were assessed. Confirmation of the viral strain was accomplished using the polymerase chain reaction (PCR) and sequencing platforms before and after culture. The cut-off for the positive/protective serum was determined at the sample dilution of 1:20 based on previous studies and from the observations on comparative testing done in the lab (44, 45).

ROC curves were constructed for the various assays and the AUC was calculated. The higher the AUC, the better the performance of the test at distinguishing between the positive and negative reports. The ROC curves for the assays were plotted with PRNT results as the state variable. From the constructed ROC curves, best threshold values were obtained and the sensitivity and specificity based on the optimal cut-offs were calculated.

The study used 125 stored, de-identified excess serum samples from the lab. Samples were randomly chosen from stored samples that were collected for an ongoing, IRB-approved study on antibody production postvaccination. Since the study used de-identified samples, there was no access to associated patient demographics. The 69 positive samples were chosen randomly from samples collected from patients 14-to-28 days postvaccination who were tested for antibodies against SARS-CoV-2. The 56 negative serum samples were selected randomly from persons who tested negative by PCR test for SARS-CoV-2 and who did a baseline antibody test prior to vaccination and had no documented history of previous COVID-19 infection or PCR-positive report. All serum samples were separated, aliquoted and stored at −80 degrees Celsius. Each individual serum sample was tested for antibodies using the PRNT method and using all six different serological assays. The results of each method were compared to the gold standard PRNT reference method.

Despite PRNT being the gold standard for detection of neutralizing antibodies, as mentioned earlier it is a complex sophisticated procedure therefore, in our study we considered combination testing of the commercial assays, which are relatively simpler and rapid with a quick TAT. To assess the performance of these assays when applied in combination, we considered parallel and series testing of the commercial assays. In parallel testing, two different assays are conducted alongside at the same time; in series testing, the tests are performed sequentially, when the first applied test shows positive.

The Genscript cPass surrogate virus neutralization test is the only FDA-approved neutralizing antibody test. It measures the ability of the antibodies to block the virus attachment to the ACE2 receptors. It is a ELISA-based method where the absorbance of the sample is inversely related to the titer of the anti-SARS-CoV-2 neutralizing antibodies. The reports are given as percentage inhibition and, based on the recent FDA recommendation, ≥30% inhibition is considered positive for SARS-CoV-2 neutralizing antibody. Therefore, Genscript cPass surrogate virus neutralization test was used in series and parallel testing with the other serological assays to evaluate whether applying a functional (qualitative test) along with the other quantitative assays improves the performances of these assays in detecting neutralizing antibodies.

All serological assays were carried out according to the manufacturers’ instructions and recommendations. Table S1 in the supplemental material shows the details of the various serological assays evaluated in this study.

Ethics approval was obtained from Department of Health (DOH) institutional review board (IRB), Abu Dhabi. All methods were carried out in accordance with relevant guidelines and regulations.

Informed consent was waived by the Department of Health (DOH) institutional review board (IRB), Abu Dhabi.

### Statistical analysis.

Sensitivity, specificity, PPV, and negative predictive value, along with the 95% confidence interval (CI) were calculated to determine the performance of each serological assays. AUC was calculated from the ROC curves for each test. Chi-square tests were used for comparing the change in sensitivity, specificity, and overall agreement using the ROC-based cut-offs. Cohen’s kappa coefficient was used to identify percentage agreement between individual assays and PRNT reports. All data were analyzed using the SPSS version 28 statistical software.

### Data availability.

All the data are available from the corresponding author S.A.M., Director of Biogenix G42 lab, and can be produced on request.
