# Supplementary material for: Serological Assays for Assessing Postvaccination SARS-CoV-2 Antibody Response
Source: Microbiol Spectr. 2021 Sep 29;9(2):e00733-21. doi: 10.1128/Spectrum.00733-21 (PMC8557923; doi:10.1128/Spectrum.00733-21)
Supplement: SUPPLEMENTAL FILE 1 — Supplemental material. Download SPECTRUM00733-21_Supp_1_seq7.pdf, PDF file, 0.1 MB [file spectrum00733-21_supp_1_seq7.pdf]

Table S1: Details of the six commercial serological assays

Assays (Type)	Method	Manufacturer	Isotype and antibody	Target protein	Run time (mins)	Manufacturer's claim of sensitivity	Manufacturer's claim of Specificity
Genscript cPass (Qualitative test)	ELISA	Genscript	IgG – NAB	RBD unit of S1	180	93.8%	99.4%
Diasorin- S1/S2 IgG (Quantitative Test)	CLIA	Diasorin	IgG- total Ab	S1/S2	35	93.9%- 99.5%	98.6% - 99.7%
Alinity IgG II (Quantitative Test)	CMIA	Abbott	IgG- total Ab	RBD unit of S1	20	89.9%- 99.7%	99.2%-99.8%
Diasorin- TrimericS IgG (Quantitative Test)	CLIA	Diasorin	IgG- total Ab	Trimeric SARS-CoV-2 spike protein	20	94.5%-99.6%	99.0%-99.7%
Roche Elecsys (Quantitative Test)	CLIA	Roche	IgG & IgM- total Ab	RBD unit of S1	20	99.91 % -100%	98.1-99.3 %
AESKULISA (Quantitative test)	ELISA	AESKU	IgG- total Ab	S1	180	95.2 %	> 99 %

Table S2: Results of six commercially available serological assays.

**Assay**		**PRNT**		**Total**
		Positive	Negative	
Genscript cPass	Positive	49	3	125
	Negative	20	53	
Diasorin-S1/S2 IgG	Positive	40	3	104
	Negative	13	48	
Alinity IgG II	Positive	52	15	104
	Negative	1	36	
Diasorin-TrimericS IgG	Positive	45	5	103
	Negative	8	45	
Roche Elecsys	Positive	48	28	96
	Negative	0	20	
AESKULISA	Positive	48	13	99
	Negative	4	34	

Table S3: Area under the curve (AUC) for the serological assays

Antibody tests	AUC	95% Confidence Interval
Genscript cPass	0.939	(0.888 - 0.990)
Diasorin-S1/S2 IgG	0.935	(0.885 – 0.986)
Alinity IgG II	0.860	(0.784 -0.936)
Diasorin-TrimericS IgG	0.953	(0.912 - 0.994)
Roche Elecsys	0.839	(0.751 - 0.927)
AESKULISA	0.928	(0.871 - 0.984)

Table S4: Comparing AUC from the ROC curves

	Genscript cPass	*Diasorin S1/S2 IgG*	*Alinity IgG II*	*Diasorin-TrimericS IgG*	*Roche Elecsys*	*AESKULISA*
Genscript cPass		0.901	0.044*	0.644	0.016*	0.742
Diasorin-S1/S2 IgG			0.075	0.574	0.031*	0.843
Alinity IgG II				0.022*	0.678	0.117
Diasorin-TrimericS IgG					0.008*	0.453
Roche Elecsys						0.052
AESKULISA						

*** $p<0.05$**

Table S5: Cohen's Kappa coefficient for agreement with PRNT results

Serological assays	Cohen's Kappa coefficient
Genscript cPass	0.638*
Diasorin-S1/S2 IgG	0.693*
Alinity IgG II	0.691*
Diasorin-TrimericS IgG	0.750*
Roche Elecsys	0.406*
AESKULISA	0.652*

*** p<0.001**
